# Long-term effect of carbohydrate reserves on growth and reproduction of *Prosopis denudans* (Fabaceae): implications for conservation of woody perennials

**DOI:** 10.1093/conphys/cov068

**Published:** 2016-02-10

**Authors:** Alejandra E. Vilela, Paola R. Agüero, Damián Ravetta, Luciana González-Paleo

**Affiliations:** CONICET-Museo Egidio Feruglio, Fontana 140, Trelew, Chubut, Patagonia, Argentina

**Keywords:** Leaf-flush, leaf mass area, non-structural carbohydrates, root carbohydrates, shoot carbohydrates, vegetative growth

## Abstract

*Prosopis denudans*, an extreme xerophyte shrub, is consumed by ungulates and threatened by firewood gathering, because it is one of the preferred species used by Mapuche indigenous people of Patagonia. In a scenario of uncontrolled use of vegetation, it is very difficult to develop a conservation plan that jointly protects natural resources and its users. We performed a field experiment to assess the impact of defoliation on growth, reproduction and stores of a wild population of *P. denudans.* We imposed four levels of defoliation (removal of 100, 66, 33 and 0% of leaves) and evaluated the short- and long-term (3 years) effects of this disturbance. Seasonal changes in shoot carbohydrates suggested that they support leaf-flush and blooming. Severely defoliated individuals also used root reserves to support growth and leaf-flush after clipping. Vegetative growth was not affected by defoliation history. Leaf mass area increased after the initial clipping, suggesting the development of structural defenses. The depletion of root reserves at the end of the first year affected inflorescence production the following spring. We conclude that *P. denudans* shrubs could lose up to one-third of their green tissues without affecting growth or inflorescence production. The removal of a higher proportion of leaves will diminish stores, which in turn, will reduce or completely prevent blooming and, therefore, fruit production the following seasons. Very few studies integrate conservation and plant physiology, and we are not aware, so far, of any work dealing with long-term plant carbon economy of a long-lived perennial shrub as an applied tool in conservation. These results might help the development of management strategies that consider both the use and the conservation of wild populations of *P. denudans*.

## Abstract (precis)

We evaluated the usefulness of long-term plant carbon economy of a xerophyte shrub as a tool in conservation. Reserves manipulation through defoliation decreased reproduction in the long-term but not growth. Root and shoot reserves can be used as indicators of how much biomass can be harvested without threatening future reproduction

## Introduction

Since ancestral times, xeric species of the desert have been consumed by wildlife and used as edible, medicinal, fodder, construction and fuel resources by different aboriginal societies (Ladio and Lozada, 2009). The worldwide-distributed genus *Prosopis* L. (Fabaceae) is one of the most important elements providing firewood, timber, edible fruits and forage for wildlife and domestic herbivores in arid and semi-arid countries (Burkart, 1976). *Prosopis denudans* Bentham, the southernmost species of the genus, is a socially significant species, producing wild edible fruits frequently used by indigenous communities of Patagonia (Ladio and Lozada, 2004). Pods are also consumed by sheep and guanaco (*Lama guanicoe*; Ladio and Lozada, 2009). These ungulates can have a dramatic impact on the vegetation of an ecosystem (Bergström and Edenius, 2003). During the summer, leaves, stems and fruits of this shrub represent >3% of guanaco diet (Baldi *et al*., 2004). During the winter, when the availability of herbaceous strata decreases, the guanaco feed mainly on the shrub strata (Puig and Videla, 1997). The introduction of sheep and the increase in the human population after the arrival of European settlers in Patagonia (∼1870) marked the beginning of ecological problems in Patagonia (Soriano, 1983). Along with the pressure posed by ungulates, firewood gathering also contributes to the threat to *P. denudans* populations, because this is one of the preferred species used by Mapuche indigenous people of Northwestern Patagonia. Dead and live wood are mixed to make the fire last longer. Live wood also goes to small industries and coal production (Cardoso *et al*., 2012). Extensive grazing and local firewood gathering also threatens other multi-stem species of the genus *Prosopis* in the Monte desert (Villagra *et al*., 2005).

Woody plants accumulate and store carbohydrate reserves in roots, stems or leaves during periods when supply exceeds demands for maintenance and growth (Kozlowski *et al*., 1991). After a severe disturbance, reserves need to be allocated to the maintenance of the surviving tissues (respiration) and the regeneration and maintenance of new stem, leaf and root tissues (Marquis *et al*., 1997; Lambers *et al*., 2002). Particularly in deciduous species such as *Prosopis*, winter respiration and the beginning of both vegetative and reproductive growth occur in the absence of photosynthesizing leaves and must be totally dependent on reserves (Loescher *et al*., 1990); therefore, an adequate reserve at the time of disturbance and a rapid redevelopment of leaf area by extensive sprouting seem critical for resilience and a rapid rebuilding of carbohydrate reserves (Landhäusser and Lieffers, 2002). The need to balance competition for external resources with tolerance for damage and disturbance, such as herbivory, fire, wind and ice, causes woody plants to invest large amounts of carbon in lignified support tissues, defense and storage (Dietze *et al*., 2014). Resprouted leaves are usually tougher than the originals. They have a higher leaf mass area (LMA) and thus, higher construction costs (Funk, 2013) but they are also better defended against external herbivores (Coley, 1983).

So far, the damage caused by wildlife, cattle and rural dwellers in Patagonia has not been quantified. Only 4.7% of arid and semi-arid Patagonia is protected from anthropogenic degradation (Burkart *et al*., 2007). The scarce information available on wild woody perennials does not allow for the development of sustainable strategies to obtain fuel plants. Physiological studies can provide mechanistic explanations for how plants respond to disturbance and therefore guide management and policy-making for plant conservation (Wikelski and Cooke, 2006; Cooke and O’Connor, 2010; Lennox and Cooke, 2014). We are not aware, so far, of any work dealing with long-term plant carbon economy of a long-lived perennial shrub as an applied tool in conservation.

In order to provide guidance for management of *P. denudans*, we performed a field experiment to assess the short- and long-term impact of green tissue loss on growth, reproduction, defense and carbohydrate stores of a wild population. After spring leaf-flush, plants were exposed throughout a growth season to four treatments of clipping (0, 33, 66 and 100% leaf removal).

We expected to decrease carbohydrate stores and change plant allocation priorities with our manipulative experiment, because after the loss of above-ground tissue, the maintenance of surviving tissues, regrowth and survival are related to non-structural carbohydrate (NSC) pools (Chapin *et al*., 1990; McPherson and Williams, 1998).

Our hypotheses were as follows.

- Shoot carbohydrate reserves (NSC_shoot_) will support the production of leaves. We predict that NSC_shoot_ will increase throughout the growth season after leaf-flush in control plants, whereas in plants under severe defoliation treatments NSC_shoot_ will remain low because of the continuous production of new leaves.
- Given the decrease or lack of current photosynthesis caused by the clipping treatments, root carbohydrate stores (NSC_root_) will sustain vegetative growth and reproduction. Therefore, we predict a depletion of NSC_root_ in severely defoliated individuals. This, in turn, will reduce vegetative growth and inflorescence production in the following years.
- Repeated clipping will induce structural defenses in leaves. Leaves flushed after clipping will show higher mass per unit leaf area (LMA) than spring leaves.

Finally, we explore the relationship between root reserves and future reproduction and we discuss the implications of short- and long-term effects of green biomass loss for the conservation of populations of *Prosopis*.

## Materials and methods

### Plant material and study site

*Prosopis denudans* is a deciduous spiny shrub, 1–2 m tall, endemic to southern Argentina (Correa, 1984). It is an extreme xerophyte and it is also the most frost hardy in the genus, growing at its southernmost limits. *Prosopis* species resprout at the beginning of spring and stay in leaf until autumn. Initiation of leaf production and cambium activity appear to be rather independent of rainfall. Most *Prosopis* species produce abundant flowers at a predictable time of year, because they bloom independently of yearly rainfall fluctuation (Mooney *et al*., 1977), responding rather to photoperiod and to the length of the growing season (Solbrig and Cantino, 1975).

This experiment was performed in the ecotone between the Monte and Patagonia deserts between September 2008 and September 2010. The Monte biogeographical province covers an extensive area, from the subtropical northern part of Argentina at 24°S to the temperate northern region of Patagonia at 44°S (Roig *et al*., 2009). The mean annual precipitation in this area is <200 mm, with a mean temperature of 13°C and an absolute minimum of −10°C. *Prosopis* is the second-most dominant genus of the Monte desert (Cabrera, 1994).

Defoliation treatments were applied to a wild population located in Estancia Santa Isabel, Rawson, Chubut, Argentina (43°24′51″ S; 65°04′46″W). Forty individuals of *P. denudans* var. *stenocarpa* Burkart of similar size and shape were selected in the field. For all selected shrubs, we measured the maximal crown diameter and the diameter orthogonal to this. With these data, we estimated shrub size by using the formulae of the ellipse (Maestre and Cortina, 2005). The mean projected area of the shrubs was 0.59 ± 0.05 m^2^. Ten shrubs were randomly assigned to each defoliation treatment. No significant differences in pre-defoliation size were found between shrubs assigned to different treatments (*F* = 0.79; *P* = 0.50).

### Defoliation treatments

To examine the short- and long-term effects of leaf removal on growth, reproduction and carbohydrate reserves, we applied the following clipping treatments (10 shrubs per treatment): (i) undefoliated control (0%-def); (ii) removal of 33% leaf area (33%-def); (iii) removal of 66% leaf area (66%-def); and (iv) removal of 100% leaf area (100%-def).

Given that a reliable quantification of damage posed by people or animals to *P. denudans* is not available, we covered all possibilities with these treatments. To achieve the levels of defoliation, removing one leaf from every three leaves in the plant was equivalent to 33%-def and two leaves from every three, 66%-def. Defoliation was completed within 2 days. The initial clipping was applied immediately after the completion of the spring leaf-flush. Leaf resprouting occurred ∼4 weeks after the initial clipping. A mid-season clipping was applied to every individual (except control) as soon as resprouted leaves of 100%-def individuals were fully expanded. Defoliation treatments were applied only during the first season of the experiment (2008).

### Morphological traits and chemical analysis

Vegetative growth was estimated at the end of the growing season (autumn 2008–2009 and 2010) in a non-destructive way, using a regression equation that significantly related the shoot length and weight (*y* = −0.4242 + 0.1*x*; *r*^2^ = 0.90). Vegetative growth was estimated with the aid of 0.2 m × 0.2 m quadrats (three subsamples per plant; 10 plants per treatment). Current year vegetative growth within the quadrat, easily recognizable by the brown colour, was measured with a measuring tape and entered in the equation previously mentioned.

The number of inflorescences per plant was estimated using the aid of 0.2 m × 0.2 m quadrats. Every inflorescence within a quadrat was counted. Three quadrats per plant were measured (three subsamples per plant). The mean of those three subsamples was expressed as the inflorescence production per unit area (0.04 m^2^ quadrat) of each plant*.* We calculated inflorescence production only with the production of inflorescences, because interannual variability in fruit production has been observed in the genus (Mooney *et al*., 1977; Salvo *et al*., 1988). Particularly in *P. denudans*, flower predation and abortion account for as much as 80% of pre-anthesis mortality (Cariaga *et al*., 2005). Given the extremely low fruit-to-flower ratio and the high between-year variability previously reported for this species, it was not possible to relate fruit production to experimental loss of leaves.

Inflorescences (10 subsamples per plant; 10 plants per treatment) were stored in plastic bags during transport to the laboratory and oven-dried at 50°C to constant weight. Inflorescence production was calculated as follows:

$$\begin{array}{l} \mathrm{Inflorescence}\;\;\mathrm{production}(g\;\mathrm{inflorescence}\;\mathrm{per}\;0.04\;m^{2})\\ \;=\mathrm{number}\;\;\mathrm{of}\;\mathrm{inflorescences}\;\mathrm{per}\;0.04\;m^{2}\\ \;\times \mathrm{average}\;\;\mathrm{weightofoneinflorescence}. \end{array}$$

The average weight of one inflorescence was calculated from 10 subsamples per plant.

During the first year of the experiment, shoots were harvested on three occasions. The first harvest was after leaf-flush and before the initial clipping, hereafter referred to as ‘initial’. The second harvest was between the initial and mid-season clippings, hereafter referred to as ‘intermediate’. The third harvest of shoots occurred at the end of the growth season, hereafter referred to as ‘final’. Spring harvest of shoots (initial) was repeated during the second and third year of the experiment. Autumn harvest (final) was repeated in the second year.

Surface roots of 0.7–1.5 cm in diameter were harvested in early autumn, at the end of the growth season, during the first and second year of the experiment. Information about the root system of *P. denudans* is not available, but a mean number of 15–30 surface roots have been described for *Prosopis flexuosa*, a coexisting species of *P. denudans* in the experimental site (Guevara *et al*., 2010). As this was a long-term experiment and sampling living plants is a destructive method, we sampled only half of the individuals (five repetitions per treatment) each year. Different individuals were sampled in the first and the second year. We did not detect any impact on the above-ground biomass.

The youngest fully expanded leaves (five subsamples per plant; 10 plants per treatment) were clipped 2–3 h after sunrise on 23 September 2008 and stored in plastic bags during transport to the laboratory. Leaf area was determined using UTHSCSA Image Tool for Windows, Version 2.02. After scanning, leaves were oven-dried until constant weight. Rehydratation of leaves was unnecessary because specific leaf area was <10 m^2^ kg^−2^ (Garnier *et al*., 2001). Specific leaf area was calculated as the ratio of leaf area to leaf weight. Leaf mass per unit area (LMA) was calculated as 1/specific leaf area according to Hanley *et al*. (2007).

Carbohydrate storage was determined as the amount of non-structural carbohydrates present in roots and shoots (NSC) by the anthrone method (Yemm and Willis, 1954). Non-structural carbohydrates include sugars, starch, fructosans and some glucosides, but not the major structural carbohydrates, such as cellulose and hemicellulose (Chiarello and Roughgarden, 1984). Extraction has been accomplished in samples by acid hydrolysis (Reynolds and Smith, 1962). Samples (500 mg) were refluxed for 1 h with 100 ml of HCl solution (10%, v/v). In this way, the starch is hydrolysed to glucose. Glucose in the hydrolysed extract can be determined colorimetrically using anthrone reagents. Anthrone solution was prepared by dissolving 200 mg of anthrone OR (Mallinckrodt Chemical Works, USA) in 100 ml of sulfuric acid solution (75%, v/v). Glass tubes filled with 2.5 ml of anthrone solution, 60 µl of NSC extract and 440 µl of distilled water were mixed and heated in boiled water for 10 min. The concentration of NSC was determined at 620 nm wavelength with a UV-270 spectrophotometer (Metrolab, Argentina), using glucose as the standard.

### Statistics

Vegetative growth, inflorescence production and NSC_shoot_ content were measured several times on the same individuals. In order to detect any overall differences between related means, we used a repeated-measures general linear model (GLM). Given that a significant treatment by time interaction was found in the analysis of NSC_shoot_ content (Table 1), we explored the simple effects of treatment and time.

**Table 1:** Repeated-measures GLM for shoot NSC concentration (as a percentage of shoot dry weight), vegetative growth (in grams of last-year shoots 0.04 m^−2^) and reproductive output (in grams of inflorescences 0.04 m^−2^) by defoliation treatment (0, 33, 66 and 100% defoliation) over time

	NSC_shoot_ (% shoot dry weight)			Vegetative growth (first–second–third year)			Reproductive output (first–second–third year)		
	d.f.	SS	*F*	d.f.	SS	*F*	d.f.	SS	*F*
Between subjects									
Defoliation treatment	3	37.95	6.08***	3	773.71	1.21^ns^	3	2321.01	4.08**
Error	36	74.85		36	7643.99		36	6824.71	
Within subjects									
Time	5	101.19	7.54*	2	93.63	0.98^ns^	2	12567.54	58.19***
Time × defoliation	15	148.51	3.69**	6	91.85	0.32^ns^	6	426.70	0.65^ns^
Error	180	482.59		72	3434.74		72	7830.94	

Abbreviations: GLM, general linear model; ns, non-significant; NSC, non-structural carbohydrate; and SS, sums of squares. Values shown are mean SS. The GLM statistics are *F*-values. *P < 0.05; **P < 0.01; ***P < 0.001. *P*-values <0.05 remained significant after Bonferroni correction by the Sidak method.

Student’s paired t-test for dependent samples was performed to check for differences in final NSC_root_ (first vs. second year) and in LMA (initial vs. mid-season clipping). The assumption of normality of residual variance was tested using Lilliefors’ test and the assumption of homogeneity of residual variance by Levene’s test. When necessary, data were transformed to attain a normal distribution. Inflorescence production was transformed using natural logarithm to meet assumptions of normality. Tukey’s HSD test was used as a *post hoc* test to examine treatment-specific effects. In all statistical tests, a *P*-value of ≤0.05 was accepted as significant, but other values are shown for descriptive purposes. To control type I error rates, we adjusted α for our analyses of shoot NSC content (Table 1) and allocation to reproduction, growth, storage and defense (Table 2 and Fig. 3), using the Bonferroni correction by the Sidak method (Sokal and Rohlf, 1995).

**Table 2:** Effect of defoliation on defense of wild *Prosopis denundans* subjected to removal of 100, 66, 33 and 0% of leaf area

Defoliation treatment	Leaf mass area (kg m^−2^)	
	Initial clipping	Mid-season clipping
0%-def	0.16 ± 0.01^ns NS^	0.16 ± 0.01^a^ ^NS^
33%-def	0.16 ± 0.01^ns NS^	0.15 ± 0.01^a^ ^NS^
66%-def	0.15 ± 0.01^A^ ^ns^	0.19 ± 0.01^b B^
100%-def	0.16 ± 0.01^A^ ^ns^	0.18 ± 0.01^b B^
Between treatments	*F*_3,36_ = 0.57 *P* = 0.55	*F*_3,36_ = 2.76 *P* = 0.06

Abbreviations: ns, non-significant between treatments; NS, non-significant between dates. For each date, a general linear model (GLM) was applied for the effects of treatment (defoliation). Lower case letters within a row indicate differences between treatments for the same date. For each treatment, repeated-measures GLMs were applied for the effects of time. Different capital letters within a column indicate significant differences between dates at the *P* < 0.05 level. *P*-values <0.05 remained significant after Bonferroni correction by the Sidak method. The GLM statistics are *F*-values.

## Results

### Shoot non-structural carbohydrate content

We evaluated the dynamics of NSC content of shoots (NSC_shoot_) over time. Given the significant time by defoliation treatment interaction (Table 1), we explored the simple effects of treatments and time to explain the interaction.

#### Treatment effect

**Figure 1:**
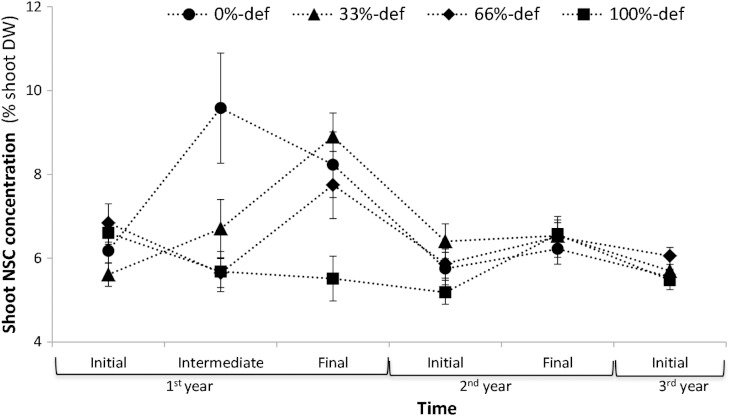
Variation of shoot non-structural carbohydrate (NSC) concentration of *Prosopis denudans* during 3 years. Shrubs were subjected to four levels of defoliation: 0, 33, 66 and 100% leaf removal. During the first year of the experiment, shoots were harvested concurrently with initial defoliation (initial), after mid-season defoliation (intermediate) and at the end of the growing season (April; final). The next year (second), shoots were harvested in early spring (October; initial) and early autumn (April; final) and the third year, in early spring (initial). Error bars indicate 1 SEM (*n* = 10). Abbreviations: def, defoliation; and DW, dry weight.

Plants subjected to different treatments showed similar initial content of NSC_shoot_ (Fig. 1; *F*_3,36_ = 2.59, not significant). Before the second clipping, NSC_shoot_ was higher in control plants (0%-def) than in defoliated plants (intermediate in Fig. 1; *F*_3,36_ = 5.36*** *P* < 0.001). At the end of the season, the NSC_shoot_ content of 100%-def individuals was significantly lower than the others (final in Fig. 1; *F*_3,36_ = 4.64*** ü*P* < 0.001). During the second and third year, there were no significant effects of treatment (Fig. 1).

#### Time effect

The NSC_shoot_ increased in control plants after spring leaf-flush expenditure (intermediate in Fig. 1; *F*_5,45_ = 4.94*** *P* < 0.001). In contrast, 100%-def plants were continuously flushing leaves throughout the season, and NSC_shoot_ remained stable and low (*F*_5,45_ = 2.27, not significant; Fig. 1). These differences between control plants and 100%-def plants accounted for the significant interaction treatment by time (Table 1). Plants in 33 and 66%-def treatments were not able to increase their NSC_shoot_ immediately after leaf-flush, like control plants, because they had to resprout partly after defoliation. Their reserve content increased after that (final; Fig. 1; *F*_5,45_ = 8.52*** *P* < 0.001 and *F*_5,45_ = 2.75** *P* < 0.01 for 33%-def and 66%-def, respectively).

### Vegetative growth and inflorescence production

**Figure 2:**
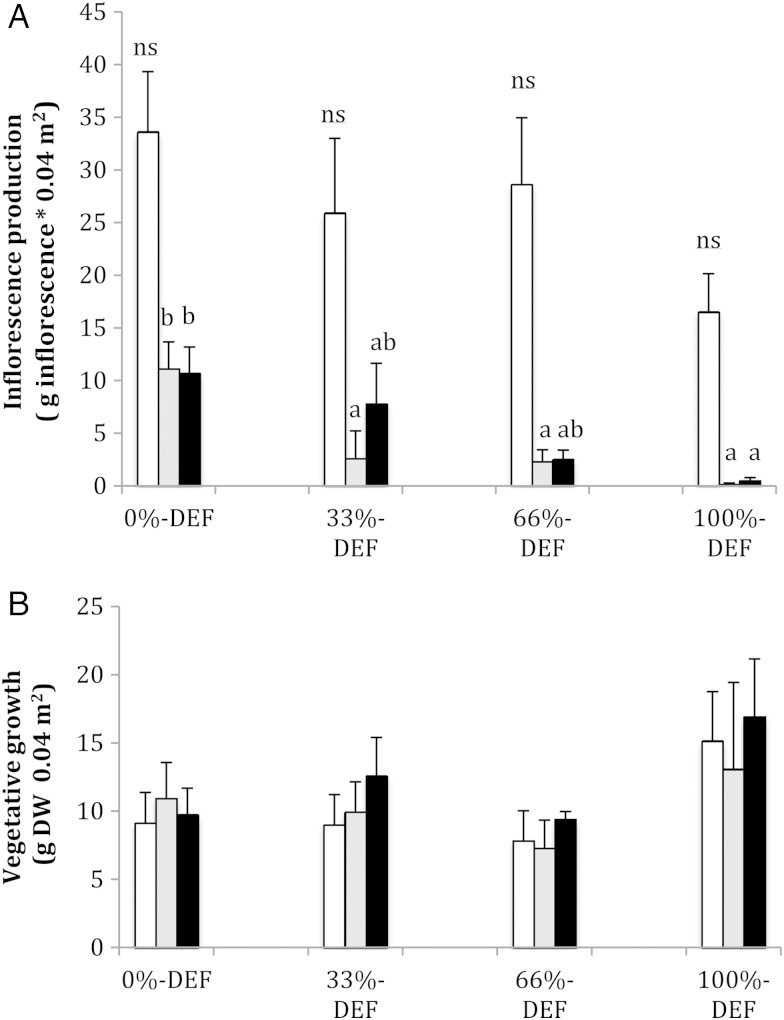
Variation of inflorescence production (**A**) and vegetative growth (**B**) of *P. denudans* during 3 years (white columns for year 1, grey for year 2 and black for year 3). Shrubs were subjected to four levels of defoliation (def; 0, 33, 66 and 100% of leaf removal). Different letters indicate significant differences among treatments for the same year. Effect of defoliation treatment on inflorescence production (A) = *F*_3,36__=_ 1.5 (not significant), 10.71*** *P* < 0.001 and 4.91*** *P* < 0.001 for year 1, 2 and 3, respectively and vegetative growth (B) = *F*_3,36__=_ 1.55 (not significant) 0.40 (not significant) and 1.29 (not significant) for year 1, 2 and 3, respectively. Error bars indicate 1 SEM (*n* = 10).

Vegetative growth was not affected by defoliation treatment or time (Fig. 2B and Table 1).

Leaves and inflorescences were produced synchronously at the beginning of spring before the application of defoliation treatments; therefore, inflorescence production did not differ between treatments during the first year (Fig. 2A). In the second year of the experiment, control plants produced more inflorescences than defoliated plants. In the third year, only individuals under 100%-def produced fewer spikes than control ones (Fig. 2A). Repeated-measures GLM indicated that there was a significant time effect (Table 1) because the production of inflorescences in the second year was lower than that of the first year for every treatment (Table 1). The significant effect of treatment corresponded to the significantly lower inflorescence production of 100%-def plants compared with that of control plants (Table 1).

### Root non-structural carbohydrate content

**Figure 3:**
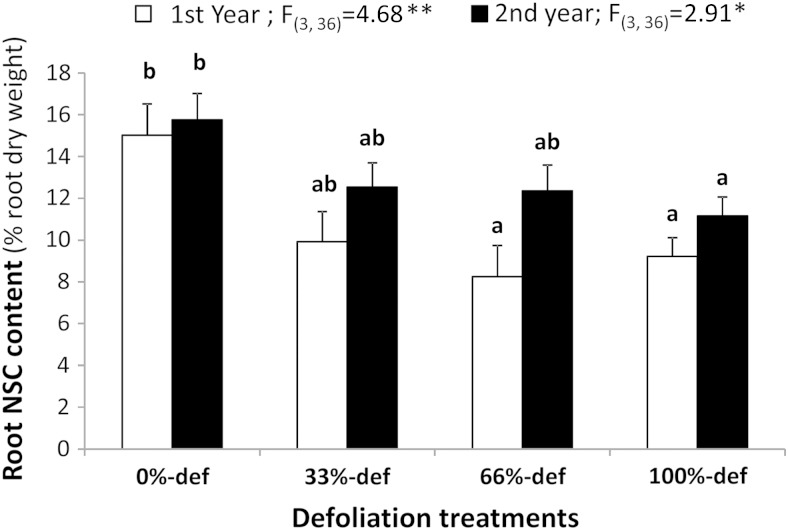
Root non-structural carbohydrate (NSC) content at the end of the first growing season, when defoliation treatments were applied (first year; open columns) and at the end of the second growing season (second year; filled columns). Shrubs were subjected to four levels of defoliation (def; 0, 33, 66 and 100% of leaf removal). Differences between years were not significant for any treatment (*P* > 0.05). Different letters indicate significant differences among treatments for the same year (*P < 0.05; **P < 0.01). Error bars indicate 1 SEM (*n* = 5).

At the end of the first growing season, NSC_root_ was significantly lower in individuals severely defoliated (66 and 100%-def) than in control individuals (Fig. 3). The NSC_root_ did not change between the first and the second year in any treatment (*P* > 0.05). The reserves of plants under 100%-def remained 30% lower than that of control plants in the second year (*P* < 0.01). The level of NSC_root_ was found to influence the inflorescence production in the following years (*y* = −2.94 + 0.31*x*; *F*_1,37_ = 4.06*** *P* < 0.001; *r*^2^ = 0.31), i.e. plants with higher NSC_root_ in year 2 produced more inflorescences in year 3 than plants with lower reserves.

### Leaf mass area

Leaf mass area of leaves initially clipped did not differ between treatments (Table 2). Leaves from individuals severely defoliated (100 and 66%-def) clipped at mid-season tended to show an increase in their LMA (*P* = 0.06; Table 2).

## Discussion

Long-lived plants, with slow life histories, present a particularly demanding challenge for plant conservation biologists because the decline of a population is difficult to assess without strong commitment to collection of demographic data (García, 2003; Marrero-Gómez *et al*., 2007). Although the most common immediately observed response to anthropogenic disturbance may be a population decline, in most instances the response to any outside force starts at the level of a species′ physiology (Coristine *et al*., 2014). Nonethless, conservation decisions may be enacted in the absence of such scientific information (Salafsky *et al*., 2002). In order to contribute to the growing need for conservation research to produce strong evidence for the consequences of human influence on wild populations, we explored how the loss of green tissue may induce changes in carbohydrate reserves of *P. denudans* and this, in turn, affects growth and reproduction in the short and long term.

Stored carbohydrates are important, particularly in arid-adapted deciduous species, for maintenance of metabolism when the duration of drought is long enough to curtail photosynthesis (McDowell *et al*., 2008), to initiate leaf area and for flowering after the winter (Chapin *et al*., 1990; Kozlowski and Pallardy, 2002; Mattson *et al*., 2008).

Seasonal changes in shoot reserves of control plants suggested that they support, at least in part, leaf-flush and blooming. After the flushing expenditure, reserves were rebuilt sooner in control plants than in those subjected to defoliation. Nonetheless, when defoliation was too severe, plants were unable to increase their levels of NSC_shoot_ during the disturbance season. This is consistent with the idea that branches are temporarily carbon autonomous, and the flushing of buds depends mainly on proximate reserves (Landhäusser and Lieffers, 2002). Despite the short-term depletion of reserves in shoot tissues of 100%-def individuals, during the following spring the shrubs did not differ in NSC_shoot_, irrespective to their defoliation history, suggesting a mobilization of stored NSC from roots to shoots, to support growth and other functions (Chapin *et al*., 1990).

Allocation of more photosynthates to roots than to shoots (highest NSC_root_ 15.8% vs highest NSC_shoot_ 9.6%) may be a safer strategy, typical of species used to disturbances (Frey *et al*., 2003; Fan *et al*., 2012). Maintaining carbohydrate reserves in the roots might be expensive (Canadell and Lopez-Soria, 1998), but in an area prone to disturbances (i.e. grazing and firewood gathering) storage in underground organs makes better sense than storage in above-ground biomass. Besides, in unproductive habitats dependence on obligate reseeding is dangerous because growth is slower and plants are more likely to be killed by disturbance without recruiting seedlings (Bond and Midgley, 2001). This is especially true for *P. denudans*, in which abortion and predation account for the destruction of 90–98% of all flowers (Cariaga *et al*., 2005).

When demand exceeds the supply of carbohydrates, root reserves are significantly depleted. Vegetative growth had a higher priority for carbohydrates because plants in different treatments of defoliation did not differ in vegetative growth. This use of root NSC to support vegetative growth has been reported in other woody perennials by Landhäusser *et al*. (2012). We were not able to find evidence of downregulation of growth to prevent depletion reserves, as has been reported for annual plants (Stitt and Zeeman, 2012).

Reductions in photosynthetic surface did not affect growth, but resulted in decreased reproduction the following season. Similar findings were recently reported for macroalgae (Geange, 2014). Our results also support the idea that a large production of flowers represents a great investment of energy (Dovis *et al*., 2014), supplied by the remobilization of NSC from roots (Mattson *et al*., 2008; Monerri *et al*., 2011). Exhaustion of carbohydrates has been identified as one of the main causes of inter-annual variability in flowering (Goldschmidt and Golomb, 1982; Crone *et al*., 2009; Monerri *et al*., 2011), a common observation reported for *Prosopis* species (Mooney *et al*., 1977; Salvo *et al*., 1988). Our contribution seems to be the first report for *Prosopis* confirming the positive role of root reserves in long-term reproduction.

Defoliation also increased allocation to defense. Leaf mass per unit leaf area has been suggested as a measure of sclerophylly (Gratani and Varone, 2004). Given than tougher leaves are better defended against external herbivores (Coley, 1983), a negative relationship between LMA and chewing herbivory has been found (Hiura and Nakamura, 2013). This trait is closely correlated with the construction cost of the leaf (Niinemets, 2001), its photosynthetic capacity, nitrogen content per unit mass and leaf lifespan (Wright *et al*., 2004).

We conclude that spring leaf-flush and blooming were supported by shoot reserves. After this expenditure of carbohydrates in spring, reserves were built up. When the supply of photoassimilates was limited by losses of green tissue, root NSC stores were remobilized to support growth, provoking a depletion of reserves at the end of the disturbance season. The next spring, shoots regained the original levels of NSC before leaf-flush, indicating remobilization from roots and further exhaustion of root stores. This depletion affected inflorescence production in the long term. Although flowering was greatly affected by depletion of reserves, vegetative growth was not, either in the short term or in the long term.

### Implications for conservation of üarid-adapted perennials

Desert shrubs constitute the major source of fuel in countries with extensive rural lands (FAO, 2008). However, in arid environments wood is limited in size and diversity, and therefore more difficult to find (Ramos *et al*., 2008). Similar to *P. denudans*, many other woody perennials in different regions of the world are at risk because of intense use (Medeiros *et al*., 2011).

In the present study, we show that root and shoot carbohydrates stores can be useful tools for conservation managers to evaluate the impact of wildlife and anthropogenic actions on the future pattern of allocation to growth and reproduction*.* These physiological traits can be used to determine levels of sustainable human use; *P. denudans* shrubs could yield up to one-third of their green tissues without affecting growth and reproductive output. In the case of removal of a higher proportion of leaves, vegetative growth will not be affected, but carbohydrate stores will be greatly diminished, which in turn will reduce or completely prevent blooming, and therefore, fruit production. The relationship among carbohydrate stores and vital rates requires further research because large populations should be monitored for long periods of time in order to determine whether a functional trait is a good predictor of demographic rate (Poorter *et al*., 2008).

## Funding

This work was funded by Agencia Nacional de Promoción Científica y Tecnológica (PICT 0598) and Consejo Nacional de Investigaciones Científicas y Tecnológicas (PIP112-2011-0100780).
